# Role of the multiple telomeric repeat arrays in integration, persistence, and efficacy of the commercial CVI988 vaccine

**DOI:** 10.1128/msphere.00142-25

**Published:** 2025-05-08

**Authors:** Luca D. Bertzbach, Yu You, Tereza Vychodil, Ahmed Kheimar, Lisa Kossak, Mohammad A. Sabsabi, Andelé M. Conradie, Benedikt B. Kaufer

**Affiliations:** 1 Institute of Virology, Freie Universität Berlin 9166, Berlin, Germany; 2 Research Unit Emerging Diseases, Leibniz Institute of Virology (LIV) 28367, Hamburg, Germany; 3 Department of Poultry Diseases, Faculty of Veterinary Medicine, Sohag University 68889, Sohag, Egypt; 4 Veterinary Centre for Resistance Research (TZR), Freie Universität Berlin 9166, Berlin, Germany; The University of Arizona, Tucson, Arizona, USA

**Keywords:** herpesvirus, CVI988, Rispens, genome integration, MDV vaccine, telomeres, vaccine effectiveness

## Abstract

Marek’s disease virus (MDV) is a highly oncogenic alphaherpesvirus that causes fatal T cell lymphomas in chickens. Oncogenic MDV strains can integrate their genome into the host telomeres of latently infected and tumor cells. This integration process is facilitated by telomeric repeat arrays (TMR) present at the ends of the MDV genome, which consist of the hexanucleotide (TTAGGG)_*n*_ that is identical to host telomere sequences. In addition, integration of the virus genome is crucial for the development of lymphomas. Live-attenuated vaccines play a vital role in protecting chickens against this deadly disease, yet our understanding of their biology remains limited. Intriguingly, the commercial gold standard MDV vaccine, the live-attenuated MDV strain CVI988, also possesses TMR at the ends of its genome. In this study, we investigated the role of the multiple TMR arrays (mTMR) in vaccine virus integration, latency, reactivation, and protection against very virulent MDV. Our data revealed that the mTMR present in CVI988 are important for virus genome integration and maintenance in latently infected cells *in vitro*. In addition, virus latency, reactivation, and vaccine efficacy were reduced in an mTMR deleted mutant compared to the wild-type vaccine. These results provide valuable insights into the biology of this important vaccine virus and shed light on the roles of the mTMR in vaccine integration, latency, and protection against very virulent MDV.

**IMPORTANCE:**

Marek’s disease virus (MDV) is an oncogenic herpesvirus and causes lethal lymphomas in chickens. The gold standard vaccine is the live-attenuated MDV strain CVI988 (a.k.a. Rispens). CVI988 is extensively used in chickens worldwide due to its high efficacy in preventing disease and lymphomas. The CVI988 vaccine harbors telomere arrays (TMR) at the ends of its genome. TMR facilitate genome integration of oncogenic MDV strains into the host telomeres. This study provides critical insights into the biology of the widely used MDV vaccine strain CVI988, demonstrating the crucial role of mTMR in viral genome integration, latency, and protection against very virulent MDV. Furthermore, our findings enhance the understanding of MDV vaccine biology and may guide future strategies to improve Marek’s disease control.

## OBSERVATION

Marek’s disease virus (MDV) is a lymphotropic alphaherpesvirus that infects chickens and causes high economic losses in the poultry industry globally (1). The widespread use of live-attenuated MDV vaccines efficiently prevents MDV-induced disease and tumors in chickens. MDV vaccines were the first to protect against virus-induced cancer and pioneered this approach (2). Currently, the live-attenuated MDV strain CVI988 is the gold standard vaccine, effectively protecting billions of chickens against disease caused by virulent field strains (3–5). We previously demonstrated that oncogenic MDV strains integrate their genomes into the telomeres of latently infected T cells and tumor cells (6). This integration is facilitated by telomeric repeat arrays (TMR) at the ends of the virus genome that are identical to the host telomere sequences (TTAGGG)_*n*_ (7, 8). Due to the genome structure, TMR are also located in the internal repeat junction (IR_L_–IR_S_) (9). There are two distinct TMR in the MDV genome: the multiple TMR (mTMR), which can vary in length and contain up to 100 repeats, and the short TMR (sTMR), which consist of a fixed number of six repeats (6–8, 10). Deletion of the mTMR did not affect the replication properties of MDV *in vitro*, while deleting the sTMR in the viral genome completely abrogated virus replication (10). Like virulent MDV, the CVI988 genome also harbors TMR at its ends (8); however, their role in the virus life cycle remained elusive. The objective of this study was to investigate (i) whether the CVI988 strain can integrate into host chromosomes, (ii) whether the mTMR facilitate vaccine integration, and (iii) whether deletion of the mTMR affects the establishment of latency and vaccine protection. Our findings provide compelling evidence that the vaccine efficiently integrates into host chromosomes and that this process is mediated by the viral TMR. This highly conserved mechanism ensures virus maintenance in latently infected hosts and allows efficient reactivation of the virus genome.

**Fig 1:**
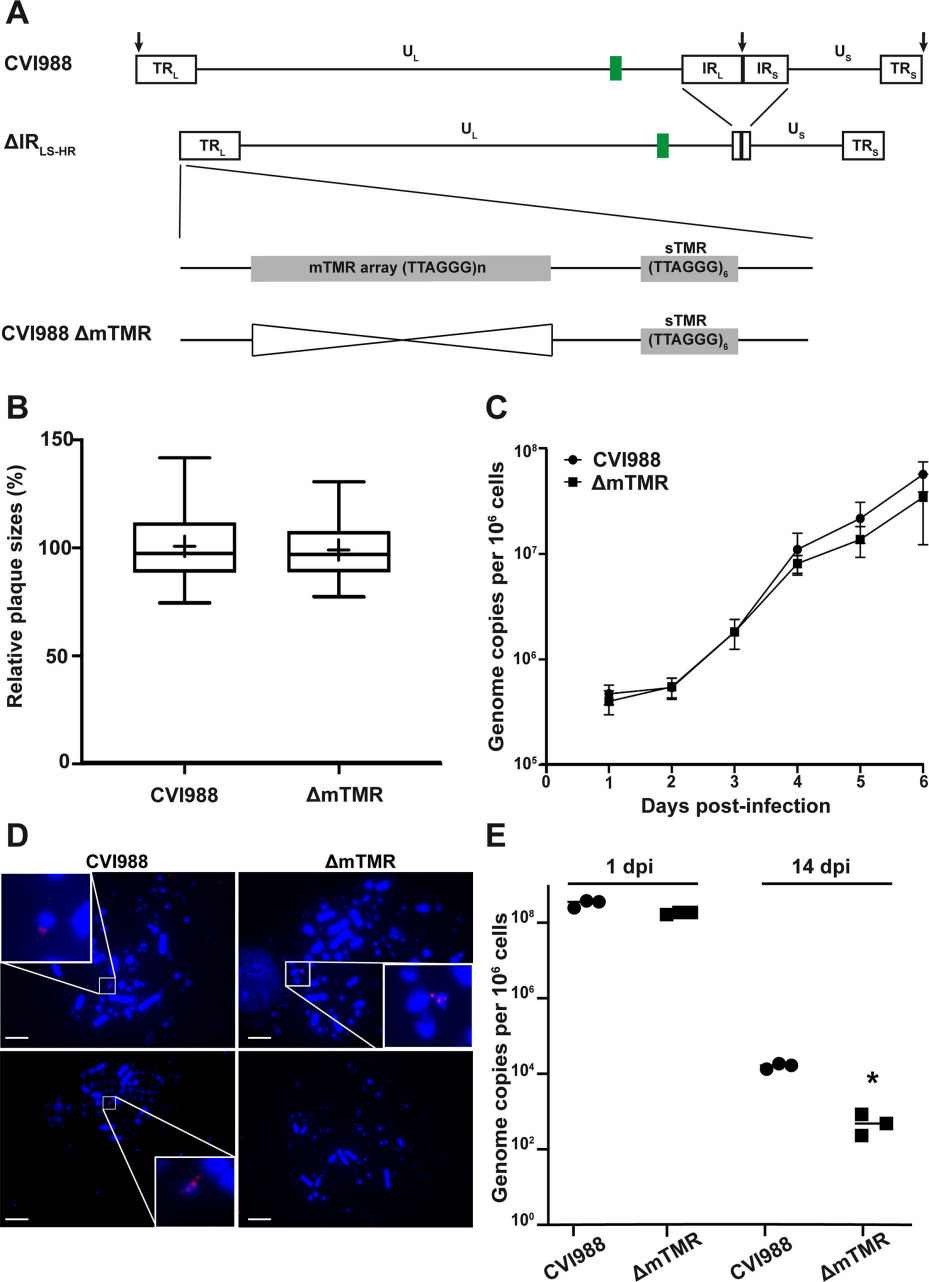
Generation and *in vitro* characterization of the CVI988 ΔmTMR mutant. (**A**) Schematic representation of the CVI988 genome with deletion of the internal repeat regions (ΔIR_LR-HR_) and deletion of mTMR (ΔmTMR). (**B**) Plaque-size assays comparing cell-to-cell spread properties of CVI988 and the ΔmTMR mutant. The box plots depict the mean plaque diameters from three independent experiments, including minimum and maximum values, a line at the median, and a “+” at the mean (*P* > 0.05, unpaired *t* test, *n* > 50 per experiment). (**C**) Multi-step growth kinetics assays comparing replication properties of CVI988 and the ΔmTMR mutant. Viral genome copy numbers were quantified using qPCR. The means of three independent experiments, along with standard deviations, are presented as copy numbers per million cells (*P* > 0.05, Mann-Whitney test). (**D**) Representative metaphase chromosomes (4′,6-diamidino-2-phenylindole [DAPI] stain, blue) showing integrated virus (Cy3 streptavidin, red) in 855-19T cells infected with CVI988 (left) and ΔmTMR (right). Scale bars represent 10 µm. (**E**) Comparison of virus maintenance after 855-19 T cell infections with CVI988 and ΔmTMR. Maintenance of viral genomes in the T cell line was assessed by qPCR analysis at 1 dpi and 14 dpi (*P < 0.05, Mann-Whitney test, *n* = 3).

We initially generated a vaccine virus lacking the mTMR (ΔmTMR) using a CVI988 bacterial artificial chromosome that encodes an enhanced green fluorescent protein reporter in its mini-F cassette (11) by two-step Red-mediated recombination as described previously (12–14) (Fig. 1A; kindly refer to the supplemental material for comprehensive details regarding all methods employed and materials utilized in this study). The resulting clones were confirmed by restriction fragment length polymorphism (RFLP), PCR, and Sanger sequencing of the deletion site. The entire genome was subsequently confirmed by next-generation sequencing (15, 16). The recombinant viruses were reconstituted in chicken embryo cells (CECs) (17), and the replication properties of CVI988 and ΔmTMR mutant were evaluated by plaque-size assays and multi-step growth kinetics (14) (Fig. 1B and C). These analyses revealed that deletion of the mTMR does not affect CVI988 replication *in vitro*, as observed for oncogenic MDV strains (6). To assess if the wild type and ΔmTMR integrate into host chromosomes, we infected the chicken T cell line 855-19 previously used to assess MDV latency and genome maintenance (18, 19). Integration was readily detectable for the wild-type vaccine at the ends of host chromosomes, while hardly any integration events were detected for the ΔmTMR mutant (Fig. 1D). While the initial infection levels were comparable between the two viruses, genome maintenance was severely impaired (~50-fold) in the absence of the TMR at 14 dpi (Fig. 1E). These data revealed that CVI988 integrates into the ends of host chromosomes and that the mTMR are important for integration and genome maintenance in latently infected cells (18).

To determine the role of mTMR in CVI988 latency and reactivation *in vivo*, we vaccinated 1-day-old chickens with 2,000 plaque-forming units (pfu) of either CVI988 (*n* = 6) or ΔmTMR (*n* = 6). Three vaccinated chickens per group were euthanized during the latent phase of infection at 14 and 28 days post-vaccination (dpv); samples were collected, and virus load was determined by qPCR (Fig. S1). During latent time points, virus load was mildly reduced in the absence of the mTMR in the blood, spleen, and isolated splenocytes (Fig. S1A through C), indicating that the establishment of latency is impaired as observed *in vitro*. To assess the reactivation properties of the two viruses, we reactivated the virus in the splenocytes. A total of 1 × 10^7^ splenic lymphocytes were seeded on a CEC monolayer and co-cultured overnight. Splenocytes were subsequently carefully removed, and the number of plaques was counted on the CECs at 4 days post-infection. Intriguingly, the number of plaques upon reactivation was drastically reduced at both 14 and 28 dpv in ΔmTMR infections (Fig. S1D). These findings indicate that deletion of the mTMR has an impact on the establishment of latency and reactivation *in vivo* and are consistent with data obtained for very virulent MDV (6, 18).

To determine the role of mTMR and/or latency in vaccine-induced protection, 1-day-old chickens were subcutaneously vaccinated with either 2,000 pfu of CVI988 (*n* = 25) or ΔmTMR (*n* = 25). The vaccinated chickens were then challenged intra-abdominally with 2,000 pfu of the very virulent plus (vv+) 686 strain at 7 dpv (20). A group of unvaccinated, 686-infected chickens (*n* = 10) served as a control. Whole blood samples were collected at indicated time points to assess vaccine and challenge replication *in vivo* (primer references: 21, 22). qPCR data revealed that ΔmTMR genome levels were reduced (Fig. S2A), especially during the latent phase of infection after 14 dpv as observed in chickens that only received the vaccine (Fig. S1A). In addition, we extracted DNA from feather pulps and dust samples (23) and quantified CVI988 genome copies by qPCR to evaluate vaccine virus shedding. Virus genome copies in feathers and dust were not significantly altered in the absence of the mTMR (Fig. S2B and C). Next, we determined the replication of the vv+ 686 strain in blood samples. Genome copies of 686 were reduced in the vaccinated groups (Fig. S2D), highlighting that both vaccines can suppress replication of the challenge virus.

**Fig 2:**
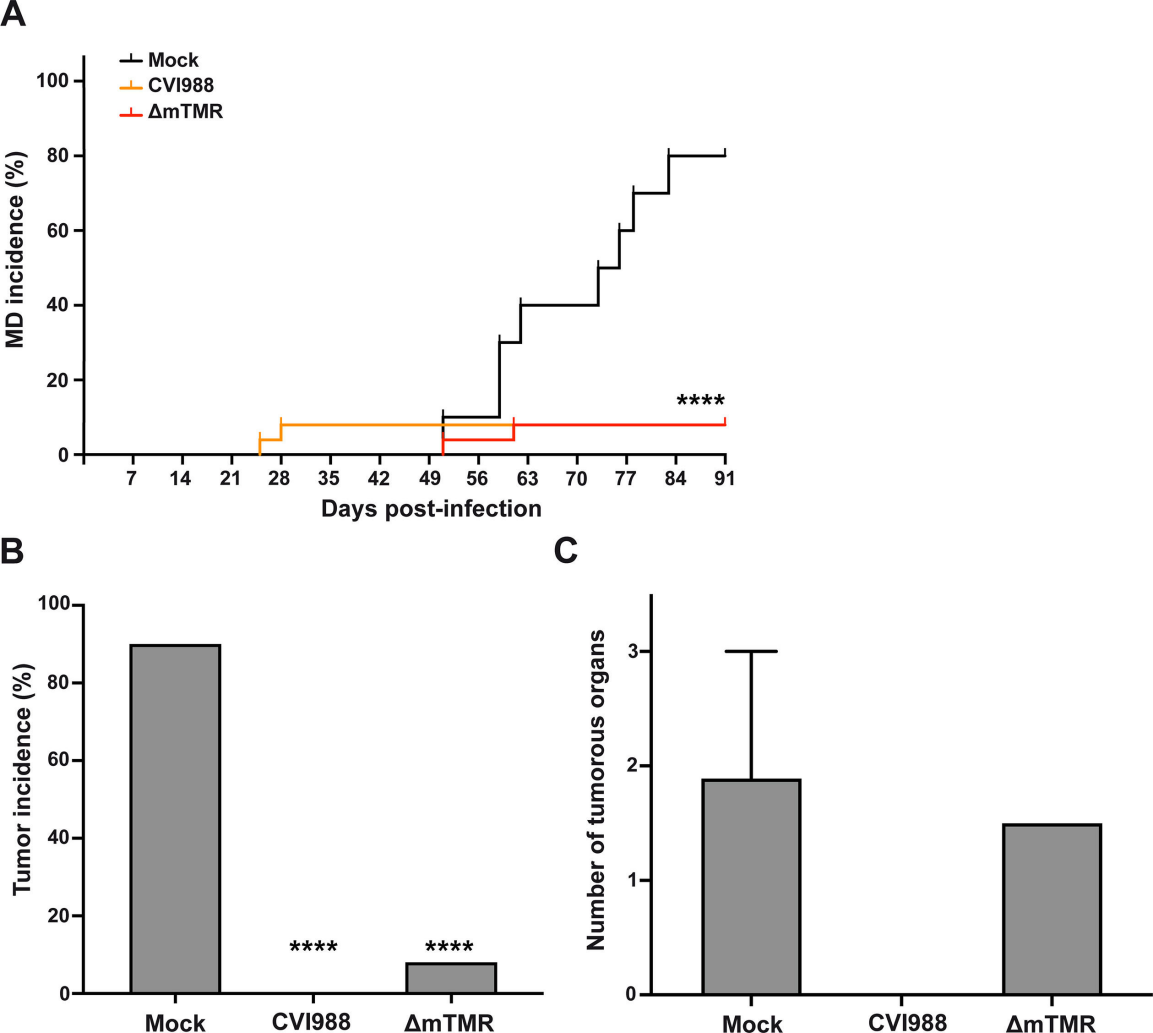
Assessment of the vaccine efficacy of CVI988 and the ΔmTMR mutant *in vivo*. (**A**) Marek’s disease incidence (clinical disease signs) in mock-vaccinated and CVI-vaccinated or ΔmTMR-vaccinated and 686-challenged chickens. A Kaplan-Meier analysis was performed to determine statistical significance (****P < 0.0001, Mantel-Cox test). (**B**) Tumor incidence of infected chickens at termination of the experiment. Results are presented as the percentage of affected chickens per group (****P < 0.0001, *χ*^2^ test). Mock group, *n* = 10; CVI988 and ΔmTMR groups, *n* = 25. (**C**) Mean number of tumor-affected organs per chicken with lymphomas in mock-vaccinated (*n* = 9) and ΔmTMR-vaccinated (*n* = 2) and 686-challenged chickens (with standard deviations [error bars]).

Beyond that, we assessed if the vaccines efficiently protect against the 686 challenge. Both vaccines drastically reduced the disease incidence (Fig. 2A) compared to the mock-vaccinated control. While no gross tumors were observed with the wild-type CVI988 vaccine, two chickens developed tumors with the ΔmTMR vaccine virus (Fig. 2B). In addition, the dissemination of the tumors in these chickens was comparable to the mock-vaccinated group (Fig. 2C). These findings highlight that the absence of the mTMR in the CVI988 vaccine impacts the establishment of latency and vaccine protection.

The present study provides valuable insights into the role of mTMR in the integration, persistence, and efficacy of the live-attenuated CVI988 vaccine. Previous research demonstrated that the related avian herpesviruses integrate into host telomeres via TMR (6, 24, 25) and suggested that the CVI988 genome may also integrate (26, 27). Our findings confirm that CVI988 efficiently integrates into host telomeres, contributing to viral persistence and optimal protection. However, the effect of mTMR deletion in CVI988 is less pronounced than observed for the recently published GaHV-3 (SB-1) vaccine lacking all of its TMR (25). SB-1 integration was more drastically impaired in the absence of its TMR, and viral persistence and vaccine protection against very virulent MDV were severely reduced. This difference could, e.g., be attributed to the residual sTMR that could not be deleted from the CVI988 genome and could still facilitate some integration via homologous recombination (10). Intriguingly, the CVI988 mutant lacking the mTMR still provided relatively good protection against the very virulent+ MDV. This effect could result from a more efficient stimulation of the immune system by the closely related CVI988 vaccine virus, compared to the more distantly related GaHV-3. Such enhanced immune activation may compensate for the lower levels of CVI988 persistence in the absence of the mTMR. Overall, these findings are consistent with previous *in vitro* and *in vivo* studies on TMR deletion viruses (6, 10, 24, 25), highlighting the need for the TMR in virus integration, latency, and vaccine protection.

## Data Availability

The virus sequences are available at GenBank under accession numbers PQ038758 (CVI988) and PQ044080 (ΔmTMR).

## SUPPLEMENTAL MATERIAL

Supplemental material msphere.00142-25-s0001.pdf Additional figures and table and detailed materials and methods.

ASM does not own the copyrights to Supplemental Material that may be linked to, or accessed through, an article. The authors have granted ASM a non-exclusive, world-wide license to publish the Supplemental Material files. Please contact the corresponding author directly for reuse.
