# Supplementary material for: Role of the multiple telomeric repeat arrays in integration, persistence, and efficacy of the commercial CVI988 vaccine
Source: mSphere. 2025 May 8;10(5):e00142-25. doi: 10.1128/msphere.00142-25 (PMC12108085; doi:10.1128/msphere.00142-25)
Supplement: Supplemental material — Additional figures and table and detailed materials and methods. [file msphere.00142-25-s0001.pdf]

Supplementary Information

Role of the multiple telomeric repeat arrays in integration, persistence, and efficacy of the commercial CVI988 vaccine.

Bertzbach et al.

Supplementary figures

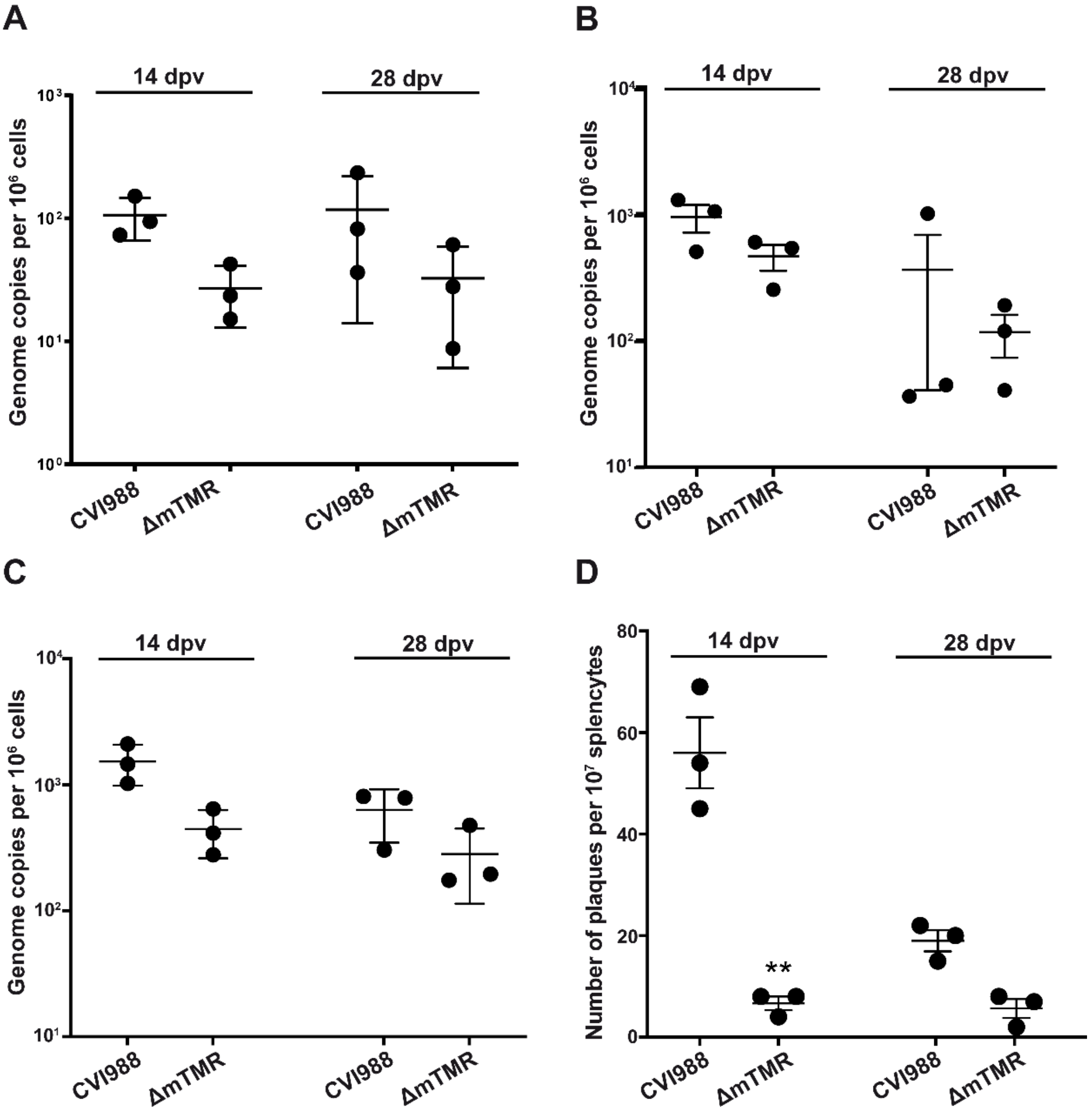

Fig. S1: Genome maintenance and virus reactivation in the absence of the mTMR.

CVI988 genome copies of the indicated recombinant viruses in (A) the spleen, (B) splenic lymphocytes, and (C) blood samples determined by qPCR at indicated time points post-vaccination (dpv). (D) Reactivation of the latent viruses from splenocytes. 10^7 splenic lymphocytes were co-cultivated with CEC and the number of plaques counted after 4 days of infection. All data are shown as dots with the means visible as horizontal bars and standard deviations as error bars (** $p < 0.01$, unpaired t-test comparing CVI988 with ΔmTMR, n = 3).

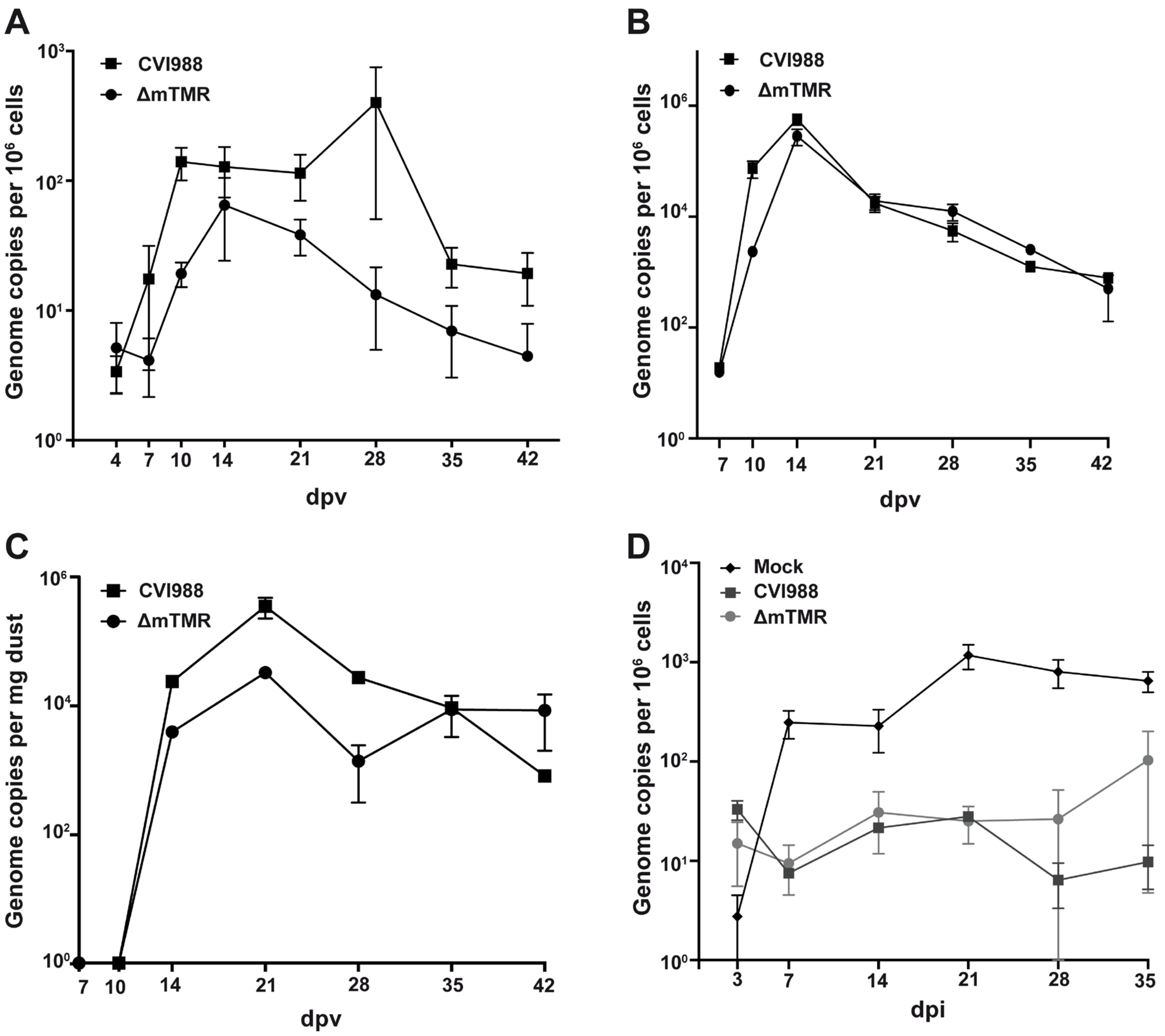

Fig. S2: Viral genome copies in the blood, feathers and dust samples from vaccinated and challenged chickens.

CVI988 genome copies for the indicated recombinant viruses in the (A) blood, (B) feather and (C) dust samples ($p > 0.05$, Mann-Whitney test). (D) 686 genome copies in blood of indicated groups challenged with the very virulent+ 686 virus at indicated time points post-infection ($p > 0.05$, Kruskal-Wallis test). n = 3 for each group and time point for panels (A-C) and n = 8 for each group and time point for data presented in panel D.

Supplementary table 1: Primers for PCR and mutagenesis, and probes used in this study.

Primer		Sequence (5' → 3')
ΔIR_{LS} mutant (Vychodil et al., 2021)	for	GTATGTGTGGGAGAAAGTATGTCGATTTTAAATGTAGTTGATATTTTTATTAGCCAAATCTAGGGATAACAGGGTAATCGATTT
	rev	TGTCAAACTTCCAGGAATACGATTTGGCTAATAAAAATATCAACTACATTTAAAATCGACGCCAGTGTTACAACCAATTAACC
ΔmTMR mutant	for	GGCTTATTTTGGATGGGGGTGGGGGGGGTGAAAATTTGGGGGGATCGGTTGGCCGCTAGTAGGGATAACAGGGTAATCGATTT
	rev	CACACTGTATAAAAAAAAATTTCGTCGAACCCCTAGCGGCCAACCGATCCCCCCAAATTTTCACCCGCCAGTGTTACAACCAATTAACC
PCR-based FISH probe 1	for	ATTACCTGGGGACAGCATGA
	rev	CACATCGTTTTGCCATGTTG
PCR-based FISH probe 2	for	CCGCTTCCTATCTCAGCAGA
	rev	TCAAGCGCTTTCTCATAGGG
PCR-based FISH probe 3	for	GAGCCAACAAATCCCCTGA
	rev	GAGGTTGGTGCTGGAATGTT
PCR-based FISH probe 4	for	CTGTTCATGTCGGAGGTCTG
	rev	GAGGGAAGCTACGGTTCAAG
PCR-based FISH probe 5	for	CCGACAATTATTGCCCCGTA
	rev	ATCTGGAAACATGTCCGACG
686 ICP4 (qPCR) (Kheimar and Kaufer, 2018)	for	CGTGTTTTCCGGCATGTG
	rev	TCCCATACCAATCCTCATCCA
	probe	FAM-CCCCCACCAGGTGCAGGCA-TAM
CVI988 pp38 (qPCR) (Baigent et al., 2016)	for	GAGCTAACCGGAGAGGGAGA
	rev	CGCATACCGACTTTCGTCAA
	probe	FAM-CCCACCGTGACAGCC-TAM
Chicken iNOS (qPCR)	for	GAGTGGTTTAAGGAGTTGGATCTGA
	rev	TTCCAGACCTCCCACCTCAA
	probe	FAM-CTCTGCCTGCTGTTGCCAACATGC-TAM

for, forward primer; rev, reverse primer; FAM, 6-carboxyfluorescein; TAM, TAMRA.

Detailed materials and methods

Cells

Chicken embryo cells (CECs) were generated from fertilized specific pathogen-free eggs (VALO BioMedia GmbH; Osterholz-Scharmbeck, Germany). The CECs were maintained in Eagle's minimal essential medium (PAN Biotech; Aidenbach, Germany), supplemented with 1% to 10% fetal bovine serum (PAN Biotech) and 1% penicillin [100 U/mL]/streptomycin [100 µg/mL] (AppliChem; Darmstadt, Germany) at a temperature of 37°C and in a 5% CO_2 environment. The reticuloendotheliosis virus (REV)-transformed chicken T cell line 855-19 was maintained in RPMI 1640 (PAN Biotech; Aidenbach, Germany) supplemented with 1% sodium pyruvate (PAN Biotech), 1% non-essential amino acids (Biochrom; Berlin, Germany), 10% fetal bovine serum and 1% antibiotics, and cultured at a temperature of 41°C and in a 5% CO_2 environment.

Viruses

The CVI988 ΔmTMR mutant was generated using a CVI988 bacterial artificial chromosome (BAC) that expresses an enhanced green fluorescent protein (eGFP) from its mini-F cassette (Bertzbach et al., 2019). The CVI988 ΔmTMR was generated as described previously (Tischer et al., 2010, Tischer et al., 2006, Vychodil et al., 2021). First, we deleted most of the internal repeat regions (CVI988 $\Delta IR_{LS\text{-}HR}$), retaining only the ends of the IR_L and IR_S regions (about 1 kbp respectively), as previously described for other herpesviruses (Vychodil et al., 2021, Denesvre et al., 2024) using two-step Red-mediated mutagenesis (Tischer et al., 2010, Tischer et al., 2006). This deletion is rapidly restored upon reconstitution and facilitates a swift manipulation of the remaining repeat regions using mutagenesis (Engel et al., 2012, Denesvre et al., 2024). Next, the entire remaining mTMR region (base pair positions 178,244 to 434 in the circular NCBI reference genome (DQ530348)) was deleted in CVI988 $\Delta IR_{LS\text{-}HR}$, resulting in ΔmTMR. The ΔmTMR mutant, in turn, also restores the $\Delta IR_{LR\text{-}HR}$, yielding a virus identical to wild-type except for the mTMR deletion. The mutants were confirmed by restriction fragment length polymorphism (RFLP), Southern blotting, and Sanger sequencing. All primer sequences are provided in Table S1. Furthermore, Illumina MiSeq sequencing with over 1000-fold coverage was conducted to validate the viruses used for in vivo research (Conradie et al., 2020). Recombinant viruses were reconstituted by transfecting CEC with purified BAC DNA using calcium phosphate transfection, as previously described (Schumacher et al., 2000). All viruses were propagated in fresh CEC, and virus stocks were frozen in liquid nitrogen and titrated before their use.

Multi-step growth kinetics

To evaluate the replication properties of CVI988 and CVI988 ΔmTMR, we conducted a quantitative PCR (qPCR)-based multi-step growth kinetics analysis, following established procedures (Vychodil et al., 2021). Here, one million CECs were infected with 100 plaque-forming units (pfu) of the two viruses. The cells were harvested every day over five days and DNA was extracted using the RTP DNA/RNA Virus Mini kit (Stratec; Berlin, Germany). qPCRs were conducted on three independent experiments to determine the MDV genome copies, using specific primers and probes for pp38 and chicken inducible nitric oxide synthase (iNOS). The virus genome copies were normalized against the chicken iNOS gene. Primer sequences are provided in Table S1.

Plaque-size assays

To determine cell-to-cell spread of CVI988 and CVI988 ΔmTMR, we performed plaque size assays following established procedures (Vychodil et al., 2021). We infected one million CECs with 100 pfu of the two viruses, used the Bioreader system (Bio-Sys; Karben, Germany) to measure plaque areas, and determined plaque diameters with the Bioreader software. We conducted three independent plaque-size assays to validate our findings.

In vitro integration assays

In order to assess the integration efficiency of CVI988 versus CVI988 ΔmTMR, we used the two viruses to infect the 855-19 chicken T cell line by co-cultivating with highly infected CEC monolayers for a period of 16 hours, as specified previously (Bertzbach et al., 2023). Subsequently, the T cells were carefully extracted, seeded into a fresh plate, and cultivated for up to 14 days. The percentages of infected T cells were then quantified using flow cytometry via the CytoFlex S flow cytometer (Beckman Coulter, Brea, CA, USA). Viral genome copies were measured at 1 and 14 dpi relative to cellular genome copies utilizing specific primers and probe for CVI988 pp38 and the cellular iNOS gene (Table S1). The integration of CVI988 was visualized in metaphase chromosomes at 14 dpi through fluorescent in situ hybridization (FISH) as described previously (You et al., 2021).

In vivo characterization of recombinant viruses

Animal experiment 1 (assessment of viral latency and reactivation)

In order to examine the impact of mTMR on CVI988 latency and reactivation in vivo, we randomly distributed one-day-old specific pathogen-free VALO chickens (VALO BioMedia) into two groups, which were housed separately. The chickens in each group were subcutaneously vaccinated with 2,000 pfu of either CVI988 (n = 6) or CVI988 ΔmTMR (n = 6). 3 vaccinated chickens per group were sacrificed at 14 and 28 days post-vaccination (dpv), and their blood and spleens were collected. To assess the reactivation properties of the two viruses, lymphocytes were obtained from the spleens using Ficoll density gradient centrifugation. The experiment was conducted in a blinded manner to minimize potential biases.

Animal experiment 2 (assessment of the vaccine protection)

In order to investigate the role of mTMR and integration in vaccine-induced protection, a total of 50 one-day-old VALO SPF chickens were subcutaneously vaccinated with either 2000 pfu of CVI988 (n=25) or CVI988 ΔmTMR (n=25). We confirmed the administered dose to the chickens through back-titration. The actual doses were comparable between the groups, with 1800 pfu for CVI988 and 1700 pfu for CVI988 ΔmTMR. The vaccinated chickens were then challenged at 7 dpv with 2000 pfu of the very virulent plus (vv+) 686 strain (Reddy et al., 2013) via intra-abdominal inoculation and the titer was also confirmed by back-titration. A group of 10 non-vaccinated 686-infected chickens was used as a control. Whole blood samples were collected at 4, 7, 10, 14, 21, 28, 35, and 42 dpv, and feathers and dust samples were collected from the chickens to assess the effects of the vaccines on 686 shedding. The experiment was performed in a blinded manner to minimize any subjective bias. The chickens were monitored daily for the onset of clinical symptoms, and if such symptoms occurred or at the end of the experiment (at 91 dpv), chickens were humanely euthanized and examined for gross tumor lesions.

Virus quantification in blood, tissues, feather follicles, and dust samples

To assess virus replication in vivo, we isolated DNA from whole blood samples using the NucleoSpin 96 Blood Core Kit (Macherey-Nagel; Düren, Germany) according to the manufacturer's guidelines. In order to evaluate virus shedding efficiency, we extracted DNA from feather pulp and dust through proteinase K treatment at 55°C overnight, followed by phenol:chloroform:isoamyl alcohol extraction and ethanol precipitation as previously described (Bello et al., 2001). Quantification of MDV genome copies was performed using qPCR, as described above.

Statistical analyses

Statistical analyses were performed using GraphPad Prism v9 (GraphPad Software, Inc.; San Diego, CA, USA). The statistics tests we used can be found in the respective figure legends. Data were considered significant if $p \leq 0.05$.

References:

BAIGENT, S. J., NAIR, V. K. & LE GALLUDEC, H. 2016. Real-time PCR for differential quantification of CVI988 vaccine virus and virulent strains of Marek's disease virus. *J Virol Methods,* 233**,** 23-36.

BELLO, N., FRANCINO, O. & SANCHEZ, A. 2001. Isolation of genomic DNA from feathers. *J Vet Diagn Invest,* 13**,** 162-4.

BERTZBACH, L. D., KOHN, M., YOU, Y., KOSSAK, L., SABSABI, M. A., KHEIMAR, A., HÄRTLE, S. & KAUFER, B. B. 2023. In vitro infection of primary chicken lymphocytes with Marek's disease virus. *STAR Protoc,* 4**,** 102343.

BERTZBACH, L. D., VAN HAARLEM, D. A., HÄRTLE, S., KAUFER, B. B. & JANSEN, C. A. 2019. Marek's Disease Virus Infection of Natural Killer Cells. *Microorganisms,* 7.

CONRADIE, A. M., BERTZBACH, L. D., TRIMPERT, J., PATRIA, J. N., MURATA, S., PARCELLS, M. S. & KAUFER, B. B. 2020. Distinct polymorphisms in a single herpesvirus gene are capable of enhancing virulence and mediating vaccinal resistance. *PLoS Pathog,* 16**,** e1009104.

DENESVRE, C., YOU, Y., RÉMY, S., VYCHODIL, T., COURVOISIER, K., PENZES, Z., BERTZBACH, L. D., KHEIMAR, A. & KAUFER, B. B. 2024. Impact of viral telomeric repeat sequences on herpesvirus vector vaccine integration and persistence. *PLOS Pathogens,* 20**,** e1012261.

ENGEL, A. T., SELVARAJ, R. K., KAMIL, J. P., OSTERRIEDER, N. & KAUFER, B. B. 2012. Marek's disease viral interleukin-8 (vIL-8) promotes lymphoma formation through targeted recruitment of B-cells and CD4+CD25+ T-cells. *Journal of virology*.

KHEIMAR, A. & KAUFER, B. B. 2018. Epstein-Barr virus-encoded RNAs (EBERs) complement the loss of Herpesvirus telomerase RNA (vTR) in virus-induced tumor formation. *Sci Rep,* 8**,** 209.

REDDY, S. M., SUN, A., KHAN, O. A., LEE, L. F. & LUPIANI, B. 2013. Cloning of a very virulent plus, 686 strain of Marek's disease virus as a bacterial artificial chromosome. *Avian Dis,* 57**,** 469-73.

SCHUMACHER, D., TISCHER, B. K., FUCHS, W. & OSTERRIEDER, N. 2000. Reconstitution of Marek's disease virus serotype 1 (MDV-1) from DNA cloned as a bacterial artificial chromosome and characterization of a glycoprotein B-negative MDV-1 mutant. *J Virol,* 74**,** 11088-11098.

TISCHER, B. K., SMITH, G. A. & OSTERRIEDER, N. 2010. En passant mutagenesis: a two step markerless red recombination system. *Methods Mol Biol,* 634**,** 421-30.

TISCHER, B. K., VON EINEM, J., KAUFER, B. & OSTERRIEDER, N. 2006. Two-step red-mediated recombination for versatile high-efficiency markerless DNA manipulation in Escherichia coli. *Biotechniques,* 40**,** 191-7.

VYCHODIL, T., CONRADIE, A. M., TRIMPERT, J., ASWAD, A., BERTZBACH, L. D. & KAUFER, B. B. 2021. Marek's Disease Virus Requires Both Copies of the Inverted Repeat Regions for Efficient In Vivo Replication and Pathogenesis. *J Virol,* 95.

YOU, Y., VYCHODIL, T., AIMOLA, G., PREVIDELLI, R. L., GÖBEL, T. W., BERTZBACH, L. D. & KAUFER, B. B. 2021. A Cell Culture System to Investigate Marek's Disease Virus Integration into Host Chromosomes. *Microorganisms,* 9**,** 2489.
